# Impact of High Glucose on Bone Collagenous Matrix Composition, Structure, and Organization: An Integrative Analysis Using an Ex Vivo Model

**DOI:** 10.3390/cells14020130

**Published:** 2025-01-17

**Authors:** Rita Araújo, Ricardo N. M. J. Páscoa, Raquel Bernardino, Pedro S. Gomes

**Affiliations:** 1 Laboratory for Bone Metabolism and Regeneration, Faculty of Dental Medicine, University of Porto, 4200-393 Porto, Portugal; rita_araujosilva@sapo.pt; 2 LAQV/REQUIMTE, Faculty of Dental Medicine, University of Porto, 4200-393 Porto, Portugal; 3 Endocrine and Metabolic Research, UMIB Unit for Multidisciplinary Research in Biomedicine, ICBAS—School of Medicine and Biomedical Sciences, University of Porto, 4050-313 Porto, Portugal; rbernanrdino@icbas.up.pt; 4 LAQV/REQUIMTE, Department of Chemical Sciences, Faculty of Pharmacy, University of Porto, 4050-313 Porto, Portugal; rnpascoa@ff.up.pt; 5 Laboratory for Integrative and Translational Research in Population Health (ITR), University of Porto, 4050-313 Porto, Portugal

**Keywords:** diabetes mellitus, bone tissue, extracellular matrix (ECM), collagen, ex vivo model, FTIR, histomorphometry, proteomics

## Abstract

Diabetes mellitus is a widespread metabolic disorder linked to numerous systemic complications, including adverse effects on skeletal health, such as increased bone fragility and fracture risk. Emerging evidence suggests that high glucose may disrupt the extracellular matrix (ECM) of bone, potentially altering its composition and organization. Collagen, the primary organic component of the ECM, is critical for maintaining structural integrity and biomechanical properties. However, definitive evidence and a comprehensive understanding of the molecular mechanisms through which high glucose impacts the ECM and collagen remain elusive. This study employed an ex vivo embryonic chicken femur model to investigate the effects of high glucose on the collagenous matrix. A comprehensive approach integrating histological evaluation, histomorphometry, ATR-FTIR spectroscopy, and proteomics was adopted to unravel structural, biochemical, and molecular changes in the ECM. Histomorphometric analysis revealed disrupted collagen fibril architecture, characterized by altered fibril diameter, alignment, and spatial organization. ATR-FTIR spectroscopy highlighted biochemical modifications, including non-enzymatic glycation that impaired collagen crosslinking and reduced matrix integrity. Proteomic profiling unveiled significant alterations in ECM composition and function, including downregulation of key collagen crosslinking enzymes and upregulation of inflammatory and coagulation pathways. High glucose profoundly disrupts the collagenous matrix of bone, weakening its structural integrity and organization. These findings emphasize the critical impact of high glucose environments on extracellular matrix composition and bone quality, offering insights into the mechanisms behind diabetic bone fragility and guiding future research toward targeted therapeutic strategies.

## 1. Introduction

The extracellular matrix (ECM) is fundamental to bone metabolism and functionality, as it orchestrates both mechanical and biological processes essential for skeletal integrity [1]. The ECM comprises inorganic components, mostly calcium phosphate, which provides stiffness and resistance to deformation, and organic components, which are primarily collagenous but also include non-collagenous proteins, lipoproteins, and glycans, contributing to elasticity and enabling the ECM to accommodate the mechanical load [2,3]. This balance between rigidity and flexibility grants bone its unique biomechanical properties. Beyond its structural role, the ECM is pivotal in regulating key biological functions deemed essential for homeostasis and tissue development [3,4]. The ECM integrates an array of local and systemic factors, which regulate cellular processes such as growth, differentiation, migration, and survival, through their role in distinct signaling pathways [5,6].

The organic matrix is primarily composed of type I collagen, the major structural protein, encoded by the COL1A1 and COL1A2 genes. Collagen is first synthesized by osteoblasts as a precursor, procollagen, that is promptly stabilized within the cell by enzymatic crosslink mediated by lysyl hydroxylases [7]. Upon its secretion into the extracellular space, procollagen undergoes cleavage to form mature collagen, which then experiences further crosslinking by lysyl oxidase. This enzyme targets lysine or hydroxylysine residues, promoting the conversion of ɛ-amino groups into reactive aldehydes, thus promoting molecular aggregation and enabling the formation of the collagen fibrillar structure [8]. This fibrillar arrangement is pivotal to the ECM organization and creates a scaffold that supports cellular adhesion through integrin-receptor signaling, which can, in turn, modulate intracellular signaling cascades that regulate subsequent cellular behaviors such as proliferation, migration, and differentiation [5]. Also, such interactions are determinants for the crosstalk between collagen and other ECM proteins (e.g., fibronectin and laminin), ensuring structural and functional integrity, and thus highlighting the importance of collagen’s role as a dynamic component that supports tissue adaptability and efficient regeneration [3].

Systemic diseases that affect bone tissue, such as osteoporosis, osteogenesis imperfecta, and diabetes mellitus (DM), are closely associated with ECM dysfunction [9,10,11]. In DM, hyperglycemia leads to the formation and accumulation of advanced glycation end products (AGEs), which are produced through non-enzymatic reactions between reducing sugars and lysine residues in proteins [5]. This glycation process alters collagen’s molecular structure, facilitating the accumulation of AGEs [12]. Clinical studies on DM patients have linked the accumulation of AGEs with the progressive deterioration of bone biomechanical properties, including increased brittleness and reduced toughness [13,14,15]. The deleterious effects of AGEs have been attributed to the non-enzymatic crosslinking with collagen fibrils, which significantly compromises collagen’s natural flexibility and leads to a stiffer, more brittle bone matrix [10]. These alterations undermine the structural and mechanical properties of bone and its overall functionality, being associated with increased fracture risk in diabetic patients [16,17,18,19].

While AGE accumulation has been recognized as a significant contributor to bone fragility, less attention has been directed toward the integrity and functionality of the collagenous network itself. Collagen, as the primary organic component of bone ECM, contributes to bone strength not only through its density but also through its precise organization, alignment, and crosslinking [2,3]. Disruptions in collagen’s molecular structure and network organization—induced by high glucose conditions—can compromise bone integrity [2,3]. Given that ECM functionality is intricately tied to the formation and maintenance of an organized collagen fibrillar pattern, focusing solely on AGE crosslinking may oversimplify the mechanisms underlying diabetic bone fragility. It is plausible that biochemical alterations in diabetes may drive both molecular and structural modifications, potentially impairing not only collagen’s ability to support bone toughness, elasticity, and resistance to microdamage. These changes may also disrupt the role of collagen in cell signaling, matrix organization, and the regulation of bone remodeling processes critical for maintaining overall bone homeostasis [2,3,20]. Consequently, investigating the collagenous matrix within diabetic conditions is critical for a comprehensive understanding of bone fragility mechanisms, providing insights that could pave the way for targeted and innovative therapeutic strategies to preserve bone quality in diabetic patients.

Accordingly, this study builds upon previous work by the research team, utilizing the ex vivo embryonic femur model, previously established to investigate the effects of high glucose conditions on bone metabolism [21]. The model preserves a high degree of cellular responsiveness to changes in the external environment due to its stage of tissue differentiation, which captures early collagen synthesis and the formation of the collagen fibrillar network [22]. This approach offers unique insights complementary to in vivo studies, which often focus on ECM properties in more mature bone tissue, where mineral composition and geometric distribution are predominant features [23]. By studying ECM organization and collagen network formation at this early development stage, this model offers a novel and invaluable perspective on how high glucose impacts ECM dynamics and collagen structure, potentially providing new insights into the understanding of diabetic bone fragility and laying critical groundwork for developing targeted interventions. Overall, the present study aims to elucidate how a high-glucose environment influences ECM composition and structure, with a focus on the collagenous network’s functional activity.

## 2. Materials and Methods

### 2.1. Organotypic Ex Vivo Femur Culture

Fertilized chick eggs (*Gallus domesticus*) were acquired from a local certified vendor. At day 11 of development, embryos were euthanized and the femora were thoroughly dissected and cleaned to remove any extraneous tissue. An organotypic culture system was established, in the air–liquid interface, using Netwel™ inserts (440 μm mesh size polyester membrane) in six-well tissue culture plates (Costar^®^, Corning, NY, USA), upon the addition of 1 mL of culture medium. In the control group, femora were cultured with alpha minimum essential medium (α-MEM) supplemented with ascorbic acid (50 μg/ mL), amphotericin B (2.5 μg/mL), streptomycin (100 μg/mL) and penicillin (100 units/mL), all Gibco^®^ (this medium contained 5.5 mM glucose, corresponding to a physiological glucose condition). In the high glucose group (GL25), femora were cultured with the control culture medium supplemented with glucose to achieve a final concentration of 25 mM. The culture plates were maintained at 37 °C, in humidified air with 5% CO_2_, for eleven days, and the culture medium was replaced daily. Upon culture period, all specimens were washed twice in phosphate-buffered saline (PBS) and either snap-frozen or fixed in 4% paraformaldehyde for further analysis [21,24].

### 2.2. Histochemical Staining

Tissue sections were prepared upon fixation, with samples embedded in paraffin and cut into 3 μm thick sections using a microtome. The sections were subsequently deparaffinized and re-hydrated to undergo a combined Alcian blue/Sirius red (AB/SR) staining protocol to assess both glycosaminoglycans- and collagen-rich areas [24]. Briefly, sections were submerged in Alcian blue solution, pH 2.5, containing 1 g Alcian blue (Merck, Darmstadt, Germany), 3 mL glacial acetic acid (Fisher Scientific, Hampton, NH, USA), and 97 mL of distilled water, for 30 min at room temperature. Later, the sections were washed in water to remove excess dye and stained with Picrosirius red solution composed of 0.1 g Sirius red (Sigma-Aldrich, Boston, MA, USA) and 100 mL saturated aqueous picric acid (Merck SA, Darmstadt, Germany), for 1 h at room temperature. After staining, sections were dehydrated through graded alcohols, cleaned, and mounted for microscopic analysis.

### 2.3. Histochemical and Histomorphometric Characterization

Histological sections were photodocumented using an Axiolab 5 microscope/Axiocam 208 camera image system (Carl Zeiss AG, Jena, Germany). The captured images were analyzed using ImageJ 1.53k open-source software for extraction of different quantitative histomorphometric indexes. For the analysis of collagen deposition, the Otsu thresholding method [25] was applied to binarize the AB/SR stained images, followed by quantitative measurements. To further characterize compositional and structural alterations in the ECM, polarized light microscopy was employed using the referred imaging system with a polarization filter. This technique enables visualization and differentiation between collagen fibrils at different maturation stages, with mature collagen fibrils exhibiting stronger birefringence, appearing red/orange, while immature fibrils display weaker birefringence, typically appearing green/yellow [26]. The polarized images were quantified by applying thresholding to separate and measure areas corresponding to mature and immature collagen. For these parameters, five samples of each group were analyzed, with five distinct regions within each sample evaluated. In addition to collagen deposition, further histomorphometric indexes—such as coherency, energy, and orientation distribution—were determined using the ImageJ plug-in OrientationJ [27].

### 2.4. Mid-Infrared Spectra (MIR) Acquisition of Tissue Samples

Upon culture period, the mid-infrared (MIR) spectra of the tissues were collected in diffuse reflectance mode using a PerkinElmer Spectrum BX FTIR Systems spectrophotometer (Waltham, MA, USA), equipped with a DTGS detector and a PIKE Technologies Gladi ATR accessory. The samples were set on the ATR accessory and compressed with a pressure of 120 N cm^−2^. The MIR spectra were recorded over a spectral range of 4000 to 600 cm^−1^, using a resolution of 4 cm^−1^ and an average of 16 scans. Each sample was analyzed in duplicate. The ATR accessory was cleaned between samples. A background spectrum was collected against air and automatically subtracted from each sample spectrum, to ensure baseline accuracy and consistency across measurements.

### 2.5. Chemometric Analysis of MIR Spectra

The chemometric models used in this study included principal component analysis (PCA) and partial least square discriminant analysis (PLS-DA) [28]. PCA was applied for initial outliers screening and visualization of sample clusters, while PLS-DA was applied for the discrimination of the samples. For the detection of outliers, only samples with scores above the threshold lines for both Hotelling’s T^2^ (weighted sum of squared scores) and Q residuals (sum of squared residuals) statistics were considered. None of the samples presented scores above the threshold lines in both graphs. All datasets were mean-centered before the application of the chemometric models. The optimization of the PLS-DA model was performed by testing different pre-processing techniques, namely standard normal variate (SNV) and the Savitzky–Golay filter (using different filter widths, polynomial orders, and first and second derivatives), both individually and in all possible combinations, using only the calibration set. To attain this, the entire dataset (58 spectra) was divided into two subsets: one for calibration using around 70% of the data (40 spectra), with the remaining 30% (18 spectra) used for validation. The division was made randomly and ensured a balanced proportion of all the classes in both sets [29]. The optimization process involved also the estimation of the best number of latent variables (LVs). The best PLS-DA model was evaluated using the total percentage of correct predictions, as well as specificity and sensitivity values obtained through the projection of the independent validation set onto the calibration set. The total percentage of correct predictions was obtained through the diagonal sum of elements contained in confusion matrices [29]. The confusion matrices also reveal which classes have the highest and lowest prediction accuracy, as well as the misclassifications of each class. The specificity and sensitivity values were calculated according to the following formulas (Equations (1) and (2)):

(1) $$Specificity=\frac{True\;negative\;values}{True\;negative\;values+False\;positive\;values}$$

(2) $$Sensitivity=\frac{True\;positive\;values}{True\;positive\;values+False\;negative\;values}$$

To identify the spectral regions with the greatest influence on the developed PLS-DA model, the regression coefficient vectors were analyzed, further allowing for the correlation of key spectral regions with potential chemical compounds present in the samples.

The chemometric analysis was performed through Matlab version 8.6 (MathWorks, Natick, MA, USA) and PLS Toolbox Version 8.2.1 (Eigenvector Research Inc., Wenatchee, DC, USA) software. In addition, ATR-FTIR metrics that are classically used to describe biophysical properties of bone tissue [30] were calculated upon the application of SNV pre-processing.

### 2.6. Proteomic Characterization

Tissue samples were processed in 0.1% triton™ X-100 for protein extraction. Liquid chromatography-mass spectrometry (LC-MS) analysis of the tissue lysates was performed in an Ultimate 3000 liquid chromatography system coupled to a Q-Exactive Hybrid Quadrupole-Orbitrap mass spectrometer (Thermo Scientific, Bremen, Germany).

A total of 500 nanograms of peptides per sample were introduced onto a trapping cartridge (Acclaim PepMap C18 100 Å, 5 mm × 300 µm i.d., Thermo Scientific, Bremen, Germany) using a mobile phase composed of 2% acetonitrile (ACN) and 0.1% formic acid (FA) at a flow rate of 10 µL/min. After a 3 min loading phase, the trap column was connected in-line with a 50 cm × 75 µm i.d. EASY-Spray column (PepMap RSLC C18, 2 µm, Thermo Scientific, Bremen, Germany) operating at 250 nL/min. Peptide separation was achieved using a gradient of solvents A (0.1% FA) and B (80% ACN, 0.1% FA) as follows: 2.5% to 10% B over 5 min, 10% to 30% B over 120 min, 30% to 50% B over 20 min, 50% to 99% B over 5 min, and held at 99% B for 10 min. The column was re-equilibrated with 2.5% B for 17 min. Data acquisition was managed via Xcalibur 4.0 and Tune 2.9 software (Thermo Scientific, Bremen, Germany).

The mass spectrometer was configured in data-dependent (dd) positive acquisition mode, alternating between a full scan (*m*/*z* 380–1580) and subsequent higher-energy collisional dissociation (HCD) MS/MS of the 10 most intense peaks from the full scan, with a normalized collision energy of 27%. The electrospray ionization (ESI) spray voltage was set to 1.9 kV. Global settings included a lock mass at *m*/*z* 445.12003, lock mass injection enabled, and chromatographic peak width (FWHM) of 15 s. Full scan parameters were a resolution of 70,000 (at *m*/*z* 200), an AGC target of 3 × 10⁶, and a maximum injection time of 120 ms. Data-dependent acquisition (DDA) parameters included a minimum AGC target of 8 × 10^3^, intensity threshold of 7.3 × 10^4^, charge state exclusion (unassigned, 1, 8, >8), peptide match preferred, isotope exclusion enabled, and a dynamic exclusion duration of 45 s. MS/MS parameters included one microscan, a resolution of 35,000 (at *m*/*z* 200), AGC target of 2 × 10^5^, maximum injection time of 110 ms, isolation window of 2.0 *m*/*z*, isolation offset of 0.0 *m*/*z*, dynamic first mass enabled, and spectrum data type set to profile.

The raw files were analyzed using Proteome Discoverer 2.5.0.400 software (Thermo Scientific, Bremen, Germany) for protein identification, using the Uniprot *Gallus gallus* database (43,711 entries). Differentially expressed proteins between the experimental groups were identified according to the following criteria: (a) each protein had to be detected in at least 2 out 3 replicates of each group; (b) at least two unique peptides per protein had to be identified, with statistical significance assessed via *p*-value adjustment using the Benjamini–Hochberg correction to control the false discovery rate (FDR), set to 0.01; and (c) proteins with a fold-change ratio ≥ 1.5 were defined as upregulated, while those with a ratio ≤ 0.667 was considered downregulated between groups [31].

Further functional analysis of the differentially expressed proteins was conducted using STRING 12.0 bioinformatics tools (https://string-db.org/ (accessed on 8 September 2024)). Proteins were classified according to Gene Ontology (GO) Biological Process annotations and REACTOME pathway annotations. FDR for pathway enrichment was calculated using the Benjamin–Hochberg method, with a significance threshold set at 0.05. In addition, specific proteins of interest were manually extracted from the dataset, and their expression ratios were calculated, in accordance with the above criteria.

### 2.7. Statistical Analysis

Statistical analyses were performed using the Statistical Package for the Social Sciences, Version 26.0 for Windows (SPSS Inc., Chicago, IL, USA), with α = 0.05 set for significance. Descriptive statistics were reported as mean and standard deviation values. A t test was performed to analyze the variables Energy and Coherency, while the Mann–Whitney non-parametric test was performed for the results of the collagen deposition area and the collagen fiber maturity deposition area.

## 3. Results

### 3.1. Histochemical and Histomorphometric Characterization

**Figure 1:**
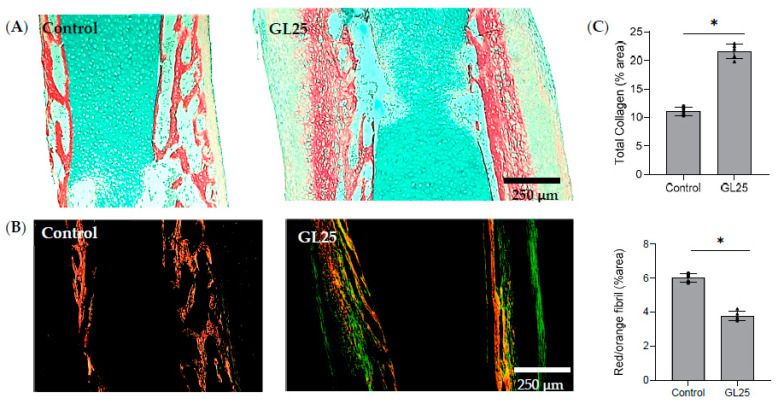
Collagen histochemical and histomorphometric characterization. (**A**)—Histochemical evaluation of total collagen content—AB/SR staining. Scale bar = 250 µm. (**B**) Histochemical evaluation under polarized light. Scale bar = 250 µm. (**C**) Quantitative assessment of total collagen (red staining area) and quantitative evaluation of mature collagen fibers (red/orange coloration, under polarized light). *—significantly different from control, *p* < 0.05.

Alcian blue/Sirius red (AB/SR) staining distinguishes collagen deposition (red stain) from the glycosaminoglycan-enriched matrix (blue stain) in the femoral mid-diaphysis. Collagen deposition is organized centripetally, forming a trabecular-like structure that progressively infiltrates the glycosaminoglycan-enriched tissue, where some areas similar to lacunae fringed by a thin layer of collagen are also observed within this matrix (Figure 1A). In the GL25 group, collagen deposition is substantially increased compared to the control group, presenting a densely packed structure without the distinct and organized trabecular-like arrangement evidenced within the control. Histomorphometric analysis, as indicated by the collagen area index, confirms that collagen deposition is around 50% higher in the GL25 group than in the control group. Histological samples were additionally observed under polarized light (Figure 1B) in order to assess collagen fibril birefringence. Comparing the control group with GL25, the control exhibits a notably more intense red coloration, indicating a higher prevalence of mature, thick collagen fibrils. In contrast, the GL25 group presents areas of green-colored fibrils within the matrix, suggesting the presence of thinner, less mature collagen fibrils. A thin marginal layer of green fibrils, correspondent to the periosteal surface, is observed across both groups.

In addition, collagen spatial organization appears more homogenous in the control group, where fibrils are predominantly aligned along the longitudinal axis of the bone. In the GL25 group, however, a multidirectional spatial arrangement of the fibrils is evident, being suggestive of disrupted structural organization under high glucose conditions (Figure 1B). Quantitative analysis of red/orange fibril deposition area shows a significant decrease of around 35% within the GL25 group, regarding the deposition area of larger, mature red/orange fibers compared to the control group (Figure 1C).

**Figure 2:**
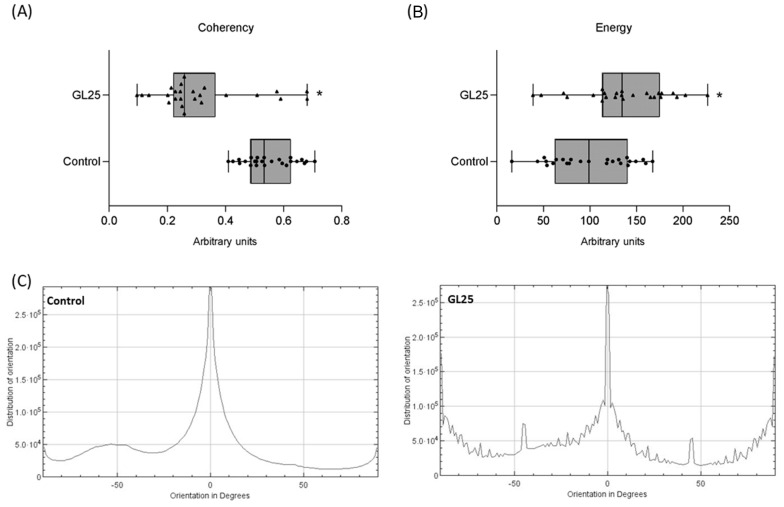
Analysis of collagen structural parameters: (**A**)—coherency, (**B**)—energy; and (**C**)—orientation distribution. *—significantly different from the control.

The structural parameters of ECM collagen reveal notable alterations in the GL25 group compared to the control. Coherency, which measures the uniformity of fibril alignment, is significantly reduced in GL25 (Figure 2A), with elevated variability observed across sections, suggesting disrupted organizational alignment of collagen fibrils. Energy, a parameter indicating the density and proximity of collagen fibrils within the ECM (Figure 2B), is significantly higher in GL25, pointing to a denser but less organized fibril structure than the control. Orientation distribution shows a broader range of fibril angles in GL25, contrasting with the control, where fibrils are predominantly aligned along a 0° orientation, consistent with the organized pattern seen in the control’s birefringence images (FIgur. Together, these findings underscore the loss of alignment and altered density of collagen fibrils within the ECM under high glucose conditions.

### 3.2. Chemometrics Analysis

**Figure 3:**
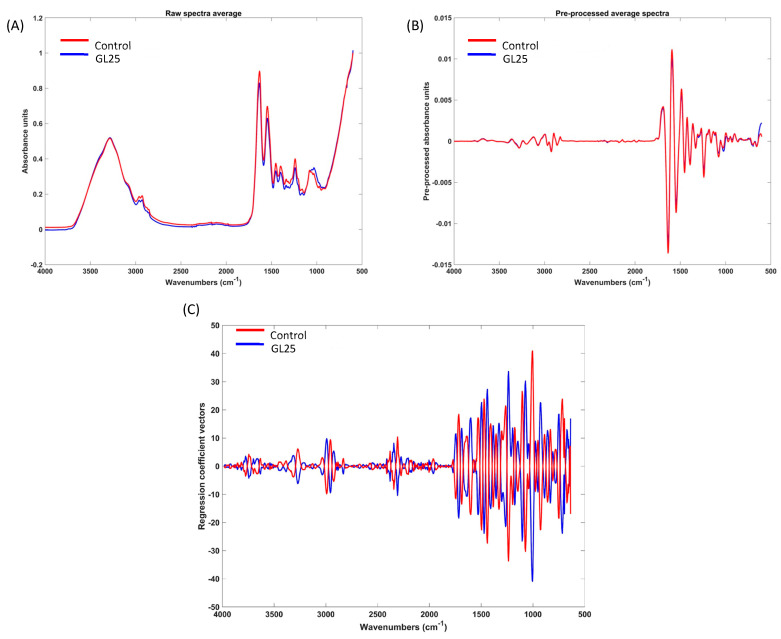
(**A**)—Raw average spectra. (**B**)—Pre-processed average spectra obtained through ATR-FTIR analysis. (**C**)—Regression coefficient vectors of the best PLS-DA model obtained from MIR spectra when pre-processed with Savitzky–Golay filter (15-point filter width, 3rd-order polynomial, and second derivative) followed by mean-centering.

As shown in Figure 3, differences can be noticed in the raw and pre-processed MIR spectra between GL25 and the control group, particularly around 100m cm^−1^. In the obtained MIR spectra, characteristic peaks are observed around 1650, 1540, and 1240 cm^−1^, which can be attributed to the C=O stretch of amide I, C-N stretching combined with N-H bending of amide II, and amide III, respectively [32,33]. The C=O stretch of amide I can be related to the amount of collagen [34]. The peaks around 1445 and 1395 cm^−1^ may be attributed to carbonate (CO_3_^2−^) and carboxylate (COO^−^) content, indicative of mineral components within the bone matrix [32,33]. The region between 1080 and 950 cm^−1^ can be connected with the amount of phosphate (PO_4_^3−^) and may also signal the presence of glycation-related carbohydrate modifications [33,35]. PCA and PLS-DA model (details provided in Supplementary Data) identified the spectral regions that contribute most to differentiate GL25 from control, specifically the interval between 1700 and 1005 cm^−1^. In addition, the PLS-DA model (Figure 3C) further establishes that the 1200–1000 cm^−1^ range is a determinant of the spectral differences between groups.

Table 1 further presents additional indexes commonly used to characterize bone tissue through ATR-FTIR analysis. The results reveal a significant increase in collagen maturity, indicated by the 1660/1690 cm^−1^ ratio, in the GL25 group compared to the control group. In addition, the collagen integrity ratio also shows a marked reduction in the GL25 group.

### 3.3. Proteomic Characterization

**Figure 4:**
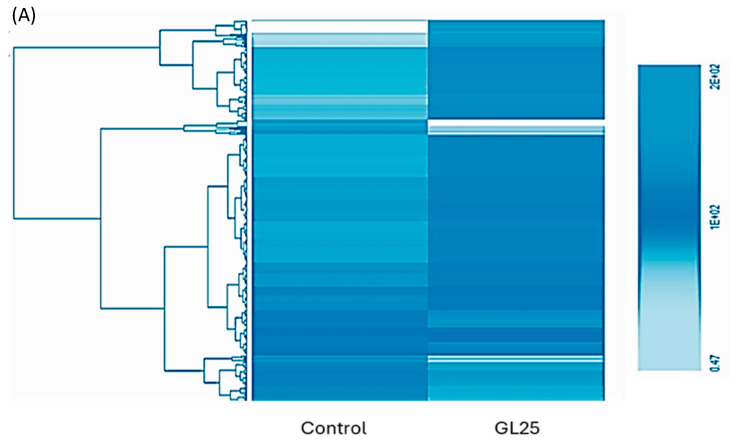

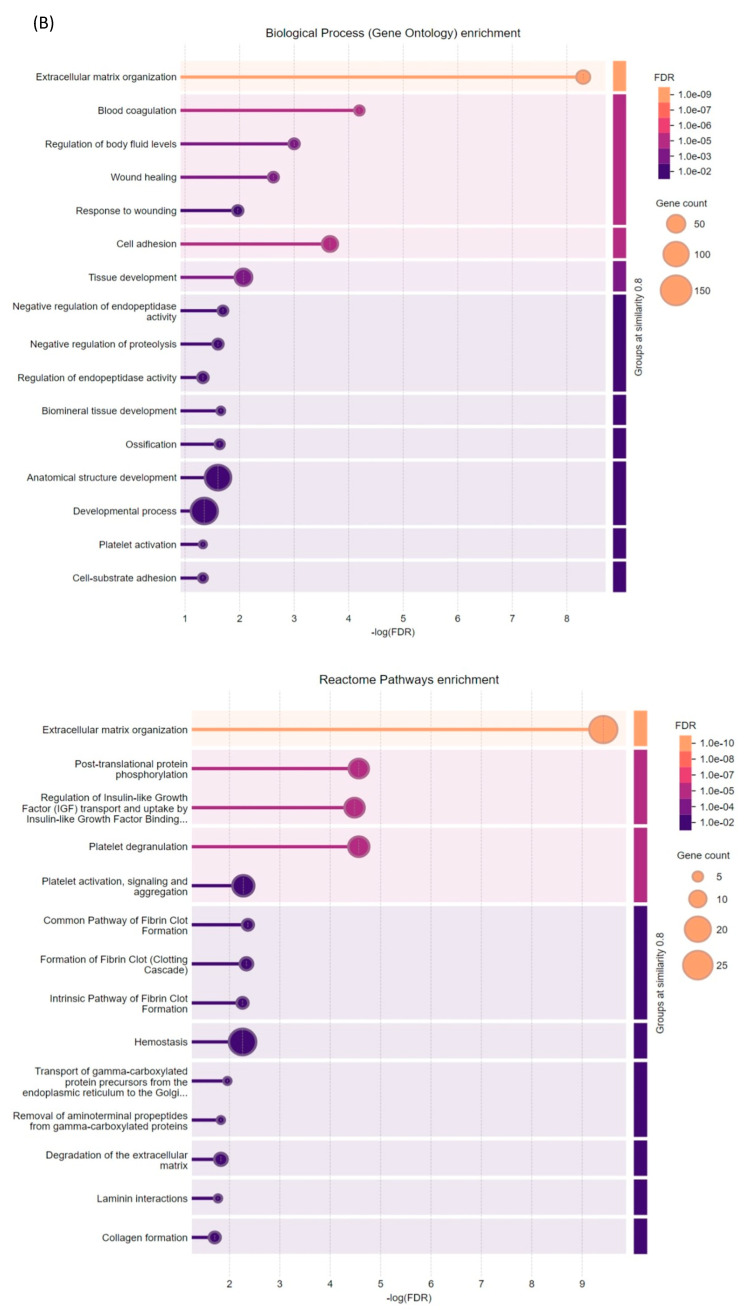
Proteome characterization. (**A**)—Heat map shows a differential expression pattern between control and GL25. (**B**)—Classification of differentially expressed proteins under GO Biological Process and REACTOME metabolic pathway.

Proteomic analysis was conducted in both groups to disclose molecular alterations related to ECM structure and function. Overall proteome overview (Figure 4A) shows a distinct pattern of expression between the control and GL25, which is evidenced by the heatmap. This difference prompted the identification of significantly upregulated or downregulated proteins, which were then classified, using STRING bioinformatics tools, under the REACTOME pathway or the Gene ontology-biological process, to further understand their relevance for the regulation of the ECM. It can be noticed that the majority of upregulated proteins are classified under metabolic pathways related to collagen assembly or maintenance (Figure 4B), in addition to processes and pathways associated with blood clot formation and coagulation process. For instance, Group GL25 is enriched in proteins classified under “Extracellular organization”, “Platelet degranulation”, “Hemostasis”, “Degradation of the extracellular matrix” and “Collagen formation”. A similar tendency is verified for the classification under GO Biological processes, since group GL25 is enriched, among others, in the following processes: “Extracellular matrix organization”, “Wound healing”, and “Response to wounding”.

Key proteins essential for ECM assembly were manually extracted from the data presented in Table 2. The analysis reveals structural and compositional alterations in the ECM, with several collagen types showing differential expression. COL10A1, COL14A1, and COL5A1 are significantly upregulated, while COL1A1 displays a tendency toward upregulation. In contrast, collagens such as COL12A1 and COL3A1 are notably downregulated.

Additionally, proteins associated with ECM functionality exhibit altered expression in the GL25 group. CCD80, FMOD, LAMB1, and VTN are upregulated, whereas PXDN is downregulated. Proteins directly involved in collagen assembly, including PLOD1, PLOD3, LOXL2, and LOXL4, are also significantly downregulated, suggesting impaired ECM stabilization under high glucose conditions.

Further, proteins linked to the blood coagulation process—such as FGA, FGB, FGG, F9, F10, SERPINC1, SERPIND1, and SERPINE2—are significantly upregulated. Proteins involved in ECM remodeling, including TGFB3 and TGFBR3, are also upregulated. Finally, inflammation markers, such as C5 and KNG1, show markedly increased expression in the GL25 group, highlighting a pro-inflammatory environment.

## 4. Discussion

The findings of this study suggest that the histopathogenesis of diabetic bone condition might result from biochemical and molecular alterations of the ECM that contribute to undermining tissue functionality. The comprehensive characterization of bone collagenous matrix demonstrates that bone tissue undergoes significant molecular and structural alterations under high glucose conditions, which are highlighted within the ex vivo embryonic chicken femur model—previously established as a representative system for addressing high-glucose-related bone alterations [21].

Histomorphometric evaluation of AB/SR-stained histological sections revealed that high glucose significantly enhanced collagen deposition, evidenced by the larger collagen deposition area in GL25 compared to the control. This elevated collagen expression is likely driven by osteoblasts and precursor cells responding adaptively to high glucose by upregulating ECM synthesis [36]. Such findings align with other diabetic models, which similarly show that high glucose conditions stimulate excessive collagen production and deposition across multiple tissues. For instance, high glucose in kidney tissues not only increases collagen production excessively but also promotes fibronectin deposition [37]. Similarly, diabetic retinopathy is characterized by compositional alterations of the ECM, with heightened collagen and fibronectin expression [38]. Although in vivo observations of glucose-induced collagen deposition in bone are limited, in vitro studies consistently demonstrate elevated production and deposition of collagen and related molecules within ECM, underscoring model-specific variations [36].

Proteomic analysis further supports the histological findings, indicating that high glucose conditions promote ECM synthesis at the molecular level. GO Biological Process and REACTOME pathway analysis reveals an upregulation in GL25 group proteins associated with “extracellular matrix organization”, suggesting increased cellular activity toward ECM synthesis and deposition. Manual data mining indicates a significant increase in core structural and functional ECM proteins, notably the trend for increased COL1A1 expression, and the significant upregulation of COL5A1, and COL14A1—both associated with the regulation of type I collagen fibrils [39]—and COL10A1, critical during endochondral ossification and ECM mineralization [40]. Moreover, the GL25 group displayed significantly upregulated levels of laminin (LAMB1), a key protein that promotes cell-ECM adhesion, supporting the cohesive integration of matrix components; vitronectin (VTN), a vital protein that fosters robust cell attachment and aids in matrix organization; fibromodulin (FMOD), a specialized proteoglycan involved in fine-tuning structural dimensions and developmental maturation within the matrix; and coil-coiled domain-containing protein 80 (CCD80), a protein integral to the formation and stabilization of the collagenous matrix [41,42].

Although the precise mechanisms by which a high glucose environment modulates collagen deposition in bone remain unclear, research in other tissues has underpinned that high glucose upregulates signaling cascades associated with blood coagulation, with increased tissue levels of coagulation factors and fibrin production contributing to ECM overproduction [43]. Proteomic analysis of the GL25 group identified enrichment of proteins under the GO Biological Process “Blood coagulation” and REACTOME pathways including “Platelet degranulation”, “Common Pathway of Fibrin Clot Formation”, “Formation of Fibrin Clot (Clotting Cascade)”, “Platelet activation, signaling and aggregation”, and “Hemostasis”, pointing to an upregulation of these events. Manual data mining revealed increased levels of fibrinogen subunits (FGG, FGA, and FGB), coagulation factors IX (F9) and X (F10), and serpins (i.e., SERPINC1, SERPIND1, and SERPINF2—coagulation and fibrinolysis regulators), indicating elevated coagulation activity. Factor X, in particular, plays a pivotal role by catalyzing the conversion of prothrombin into thrombin, a key step that promotes fibrin formation and triggers ECM deposition through TGF-β-dependent pathways [44,45]. Thrombin-activated TGF-β is a major ECM regulator, stimulating the synthesis of collagen and other matrix components while modulating cellular processes such as proliferation, differentiation, and migration [46]. Increased TGF-β signaling in GL25 was supported by proteomic evidence showing elevated levels of TGFB3 and TGFBR3.

Activation of the coagulation cascade is likely triggered by high-glucose-induced inflammatory responses, previously demonstrated by the research team using this experimental model [21]. Fibrin and thrombin also act as potent pro-inflammatory mediators, triggering the production of cytokine and chemokines, further amplifying the synthesis of ECM components. Proteomic evidence further supports this inflammatory activation, as indicated by the increased levels of kiniogen 1 and complement factor C5. Elevated kininogen 1 reflects kinin–kallikrein system activation, leading to the production of bradykinin—a potent pro-inflammatory peptide [47]. Similarly, the upregulation of complement factor C5, which is cleaved into the pro-inflammatory fragment C5a, highlights active complement system involvement, contributing to cell activation and cytokine release [48]. Collectively, these findings confirm a heightened pro-inflammatory environment under high glucose conditions, likely exacerbating ECM remodeling and collagen deposition.

Despite the increased collagen deposition in high glucose conditions, the ECM’s quality and functional characteristics appear compromised. The histomorphometric evaluation demonstrated disrupted fibrillar organization, evidenced by altered fiber orientation and increased separation indexes in the GL25 group. Fibril orientation variability and pronounced separation were observed, with a reduced coherency parameter indicating disorganized fibril alignment, and an elevated energy parameter suggesting increased spacing. This disorganized collagen arrangement, despite the enhanced deposition, indicates compromised structural integrity. Additionally, polarized light histochemical analysis revealed fewer mature fibrils in the GL25 group, with color changes correlating with inadequate fibril diameter and impaired assembly [8,49], indicating that while collagen synthesis is upregulated, the resulting ECM lacks the organization required for optimal function.

ATR-FTIR analysis confirmed the altered chemical profile of the GL25 group, showing glycation-related peaks within the amide I band, indicating the presence of non-enzymatic reactions with the formation of AGEs such as carboxymethyl lysine (CML) and carboxymethyl arginine (CMA) [5,12,50,51]. These AGEs, products of the carbonyl group reacting with lysine or arginine side chains, interfere with collagen crosslinking and assembly, impairing structural integrity and contributing to reduced biomechanical properties and increased tissue brittleness [5]. Discriminant spectral regions in PLS-DA and PCA analyses, particularly 1200–1000 cm^−1^ and 1100–900 cm^−1^, previously associated with the glycation of collagen [52], further highlighted glucose-collagen interactions, reinforcing these biochemical ECM changes.

Additional significant variations in key FTIR ratios provided valuable insights into the state of collagen within the ECM, particularly regarding maturity, integrity, and crosslinking. The collagen maturity ratio (1660/1690 cm^−1^) was significantly increased in the GL25 group, suggesting a shift toward pathological modifications, likely driven by the accumulation of advanced glycation end-products (AGEs) and alterations in crosslinking. In contrast, the collagen integrity ratio (1240/1450 cm^−1^) was significantly reduced, reflecting disruptions in collagen’s molecular structure and secondary organization. These alterations in the GL25 group suggest that collagen glycation and AGE accumulation influence the ratios’ outcomes through non-enzymatic modifications, rather than through natural enzymatic crosslinking processes, in line with previous in vitro [51] and in vivo [52] studies. Unlike enzymatic-mediated crosslinks [53] that are essential to fibrillar assembly and intermolecular collagen interactions [54], non-enzymatic glycation-derived crosslinks can stiffen the ECM, reducing resilience and dynamic adaptability in bone tissue [19,55]. These spectral findings align with histological observations, indicating disrupted collagen organization and impaired fibrillar structure under high glucose.

Proteome analysis highlighted additional molecular changes likely contributing to the altered ECM assembly and function. Despite an upregulation in ECM formation proteins, key post-translational processes essential to ECM structure and stability were compromised. High glucose exposure led to a decreased expression of collagens critical for fibrillar organization, including COL12A1 and COL3A1, which regulate fibril diameter, alignment, and structure stability in the ECM [56]. This decrease aligns with histomorphometric data from polarized light evaluation, showing fiber disorganization, decreased alignment, and abnormal fibril diameter.

Proteins essential for collagen maturation, specifically procollagen-lysine 2-oxoglutarate 5-dioxygenases (PLODs) and lysyl oxidases (LOXs), were significantly downregulated in the GL25 group. PLODs catalyze the post-translational hydroxylation of lysine residues in procollagen, an essential step for the subsequent collagen crosslinking. Following this, LOXs perform oxidative deamination on both lysine and hydroxylysine residues, generating reactive peptidyl aldehydes that promote intramolecular and intermolecular crosslinks within and between collagen fibrils, creating a stable and resilient collagen matrix [5].

In the GL25 group, PLOD1 and PLOD2 were significantly downregulated. While the signaling mechanisms underlying this downregulation under high glucose remain unclear, in vitro studies suggest a correlation with the accumulation of AGEs [57]. In addition, LOXL2 and LOXL4 expression were also significantly reduced in the GL25 group. Previous findings demonstrated high expression of LOX and LOXL1 (other members of this family) in normal bone but significant downregulation under high glucose [58]. This study’s unique downregulation of LOXL2 and LOXL4 may reflect the distinct temporal expression patterns, as these isoforms play a key role during the mineralization phase of endochondral ossification [59]. This phase aligns with the developmental timing of the used embryonic model, suggesting that high glucose conditions disrupt ECM maturation by targeting LOX isoforms critical for early bone mineralization. Furthermore, PXDN, an enzyme responsible for catalyzing the formation of sulfilimine bonds between collagen IV molecules, was also downregulated under high glucose conditions. PXDN facilitates the formation of unique crosslinks in collagen IV, which are critical for stabilizing basement membranes and providing tensile strength to connective tissue [60].

The downregulation of PLOD1, PLOD2, LOXL2, LOXL4, and PXDN likely results in impaired collagen crosslinking and inadequate ECM stabilization, compromising the structural integrity of the developing bone matrix. This interpretation is consistent with histomorphometric data obtained from polarized light evaluations, which revealed disorganized collagen fibers, reduced fibrillar alignment, and diminished crosslinking density. Such ECM abnormalities are indicative of weakened bone structure and resilience, underscoring the impact of high glucose exposure on the molecular mechanisms governing collagen maturation and bone strength.

## 5. Conclusions

This study highlights the profound impact of high glucose conditions on the extracellular matrix, particularly the collagen network within bone tissue. The ex vivo chicken embryonic femur offers a robust model to investigate the organization and dynamics of the collagenous matrix during the early stages of bone formation. This approach has aimed to elucidate the impact of hyperglycemic conditions on the early extracellular matrix deposition assembly, addressing a gap in research that predominantly focuses on the later stages of bone maturation and mineralization processes. In this frame, we demonstrated that high glucose disrupts collagen organization, impairs crosslinking, and alters the biochemical and molecular composition of the ECM. Histomorphometric analysis revealed disorganized collagen fibril architecture displaying poor fibrillar assembly and maturation levels, while ATR-FTIR spectroscopy identified increased non-enzymatic glycation—further confirmed by the development of the chemometric analysis model—and reduced collagen integrity. Proteomic profiling further confirmed significant changes in ECM-associated pathways, including reduced enzymatic crosslinking activity and increased pro-inflammatory and coagulation signals. To the best of the author’s knowledge, no previous comprehensive characterizations of bone tissue cultured ex vivo have been conducted, allowing an overview of the global impact of high glucose exposure on distinct interacting signaling pathways.

These findings collectively emphasize the detrimental effects of high glucose on ECM composition and functionality, contributing to compromised bone quality. Therefore, the present results leverage the understanding of the bone diabetic condition by uncovering the potential therapeutic targets, especially those related to pro-coagulants, that to date have not been explored within the context of the bone diabetic condition.

## Figures and Tables

**Table 1:** Bone indexes determined by ATR-FTIR analysis. Results are presented as mean± standard deviation. Results were considered to be significant for *p* < 0.05.

	Control	GL25	*p*-Value
*Collagen maturity*	17.93 ± 3.66	23.72 ±2.73	0.04
*Collagen integrity*	0.0080 ± 0.002	0.0056 ± 0.0015	<0.0001

**Table 2:** Abundance ratio (GL25/control) of relevant proteins.

*Protein*	*Abundance Ratio (GL25/Control)*
COL12A1	0.6
COL10A1	2.6
COL14A1	2.8
COL1A1	1.3
COL5A1	1.8
COL3A1	0.6
COL12A1	0.6
LOXL2	0.040
LOXL4	0.5
PLOD1	0.62
PLOD2	0.62
CCDC80	2.2
FMOD	1.8
LAMB1	1.8
VTN	1.9
PXDN	0.5
FGA	7.8
FGB	4.1
FGG	6.1
F9	10.03
F10	3.3
SERPINC1	2.6
SERPIND1	3.3
SERPINE2	3.2
TGFB3	8.1
TGFBR3	1.8
C5	7.8
KNG1	5.1

Ratio adjusted *p*-value < 0.05. COL12A1—Collagen alpha-1(XII) chain, COL10A1—Collagen alpha-1(X) chain, COL14A1—Collagen alpha-1(XIV) chain, COL1A1—Collagen alpha-1(I) chain, COL5A1—Collagen alpha-1(V) chain, COL3A1—Collagen alpha-1(III) chain, LOXL2—Lysyl oxidase homolog 2, LOXL4—Lysyl oxidase homolog 4, PLOD1—Procollagen-lysine, 2-oxoglutarate 5-dioxygenase 1, PLOD2—Procollagen-lysine,2-oxoglutarate 5-dioxygenase 2, CCDC80—Coiled-coil domain-containing protein 80, FMOD—Fibromodulin, LAMB1—Laminin subunit beta-1, VTN—Vitronectin, PXDN—Peroxidasin homolog, FGA—Fibrinogen alpha chain, FGB—Fibrinogen beta chain, FGG Fibrinogen gamma chain, F9—Coagulation factor IX, F10—Coagulation factor X, SERPINC1—Antithrombin-III, SERPIND1—Heparin cofactor 2, SERPINE2—Glia-derived nexin, TGFB3—Transforming growth factor beta-3, TGFBR3—Transforming growth factor beta receptor type 3, C5—Complement component C5, KNG1—Kininogen-1.

## Data Availability

Data available on request.

## Supplementary Materials

The following are available online at https://www.mdpi.com/article/10.3390/cells14020130/s1, Figure S1: Raw and MIR spectra, Figure S2: PCA scores, Figure S3: Regression coefficient vectors of the best PLS-DA model; Table S1: Confusion matrix for the best PLS-DA model, Table S2: Sensitivity and specificity values calculated the best PLS-DA model, References [28,32,33,34,35,61,62,63] are cited in the Supplementary Materials.
