# Supplementary material for: Impact of High Glucose on Bone Collagenous Matrix Composition, Structure, and Organization: An Integrative Analysis Using an Ex Vivo Model
Source: Cells. 2025 Jan 17;14(2):130. doi: 10.3390/cells14020130 (PMC11764406; doi:10.3390/cells14020130)
Supplement: Supplementary file 1 [file cells-14-00130-s001.zip › cells-3413388-supplementary.pdf]

Supplementary data

1. Mid-infrared spetra acquisition of the tissue samples

The mid-infrared spectra of femora from both experimental were collected in diffuse reflectance mode using a PerkinElmer Spectrum BX FTIR Systems spectrophotometer (Waltham, USA) that has a DTGS detector and a PIKE Technologies Gladi ATR accessory. The samples were set on the ATR accessory and compressed with a pressure of 120 N cm^{-2}. The spectra acquired were comprised within 4,000 to 600 cm^{-1}, using as resolution 4 cm^{-1} and from the average of 16 scans. Each sample was analysed in duplicate. The ATR accessory was cleaned from sample to sample. The background was collected against air on the beginning of the experiment.

2. Chemometrics analysis of MIR spectra

The chemometric models used in this study were principal component analysis (PCA) [61] and partial least square discriminant analysis (PLS-DA). PCA was applied for outliers screening and cluster formation visualisation. PLS-DA was applied for the discrimination of the samples. No outliers were observed through the analysis of Hotelling's T2 (weighted sum of squared scores) and Q residuals (sum of squared residuals) statistics (data not shown). All data set was mean centered before the application of the chemometric models. The optimization of PLS-DA model was performed testing different pre-processing techniques, namely standard normal variate (SNV) and Savitzky-Golay filter (using different filter widths, polynomial orders and first and second derivatives), individually and in all possible combinations using only the calibration set. To attain this, the entire set (58 spectra) was divided into two sets: one for calibration using around 70% of the entire set (40 samples) and other for

validation using the rest 30% of the entire set . The division was made randomly and ensuring a balanced proportion of all the classes in both sets [28]. The optimization process involved also the estimation of the best number of latent variables (LV). The best PLS-DA model was evaluated using the total percentage of correct predictions, specificity and sensitivity values obtained through the projection of the independent validation set onto the calibration set. The total percentage of correct predictions were obtained through the diagonal sum of elements contained in confusion matrices [28]. The confusion matrices reveal also which classes were best or worst predicted as well as the misclassifications of each class. The specificity and sensitivity values were calculated according to the following formulas (eq. 1 and 2) :

$$Specificity = \frac{True\ negative\ values}{(True\ negative\ values + False\ positive\ values)} \quad \text{(eq. 1)}$$

$$Sensitivity = \frac{True\ positive\ values}{(True\ positive\ values + False\ negative\ values)} \quad \text{(eq. 2)}$$

To understand the spectral regions with the highest importance for the developed PLS-DA model, the regression coefficient vectors were analysed. This analysis is also important to relate the more important spectral regions with possible chemical compounds present in the samples.

The chemometric analysis was performed through Matlab version 8.6 (MathWorks, Natick, USA) and PLS Toolbox version 8.2.1 (Eigenvector Research Inc., Wenatchee, USA) software.

3. Unsupervised analysis: spectral analysis and Principal Component Analysis

The average raw MIR spectra (Fig. S1) as well as the average pre-processed MIR spectra (Fig. S1) using the pre-processing technique for the best PLS-DA (specifically, Savitzky-Golay filter with 15 points filter width, 2nd polynomial order and second derivative, followed by mean-centering) of all samples are shown in Fig. S1. The acquired raw MIR spectra are similar to bone MIR spectra already provided in the literature [e].

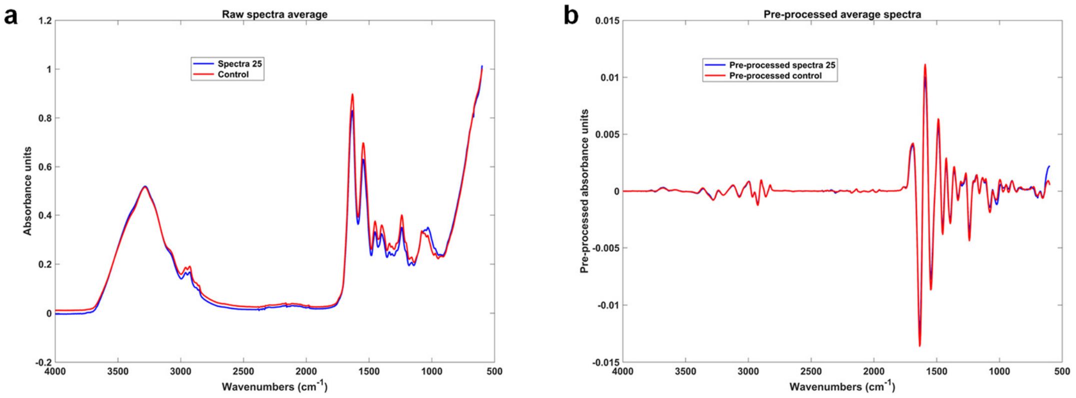

Fig. S1 . Raw spectra (a) and pre-processed with Savitzky-Golay filter (15 points filter width, 2nd polynomial order and second derivative) followed by mean-centering (b) average MIR spectra.

As can be observed, there are some differences between the raw and pre-processed MIR spectra of these two classes, particularly around 1,000 cm^{-1}.

Regarding the obtained MIR spectra, the peaks around 1,650, 1,540 and 1,240 cm^{-1} can be attributed to the C=O stretch of amide I, C-N stretching plus N-H bending of amide II and amide III, respectively [32,33]. The C=O stretch of amide I can be associated with the amount of collagen [34]. The peaks around 1,445 and 1,395 cm^{-1} may be related with CO_3^{2-} and COO^- content [32,33. The signal between 1,080 and 950 cm^{-1}

can be connected with the amount of PO_4^{3-} and with carbohydrates glycosylation [32,33,35].

As abovementioned, no outliers were detected through PCA. Additionally, PCA was used to verify the presence of clusters for the different classes. This was visualised through the scores map of different principal components (PCs) against each other, considering the pre-processing technique (application of Savixtky-Golay filter using 15 points of filter width, 2nd polynomial order and second derivative) used in the best PLS-DA model. The respective PCA model comprised 4 PCs, which captured 94.5% of total variance. As shown in Fig. 2, there is a slight tendency for differentiation between the two groups of samples.

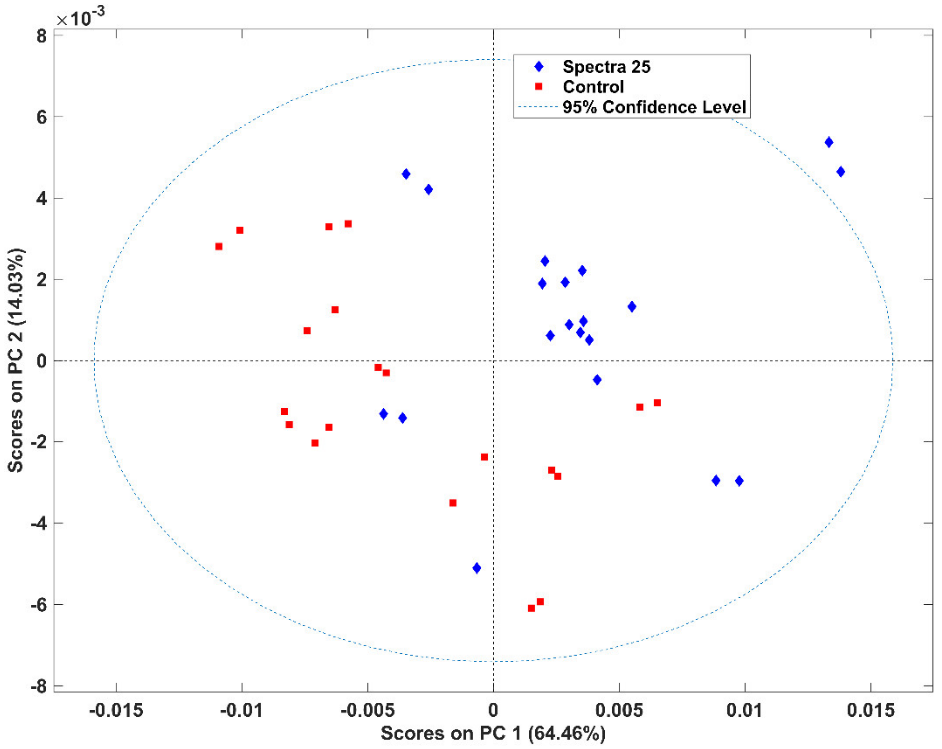

Fig. S2 . PCA scores of PC1 versus PC2 considering the pre-processed MIR spectra (application of Savixtky-Golay filter using 15 points of filter width, 2nd polynomial order and second derivative, followed by mean-centering) of the entire set.

In fact, the samples belonging to the spectra 25 group tend to have positive scores in PC1 while samples from the control group tend to have negative scores in PC1. This was expected due to the spectral differences seen in Fig. S1.

Therefore, these results further support the need of performing a supervised analysis to determine if it is possible to effectively discriminate between these two classes.

4. Supervised analysis: Partial least squares discriminant analysis-PLS-DA

In this context, PLS-DA was the chemometric model selected for the discrimination of the samples. The best PLS-DA model was obtained using 3 LV and Savitzky-Golay filter (15 points filter width, 2nd polynomial order and second derivative) followed by mean-centering as the pre-processing technique. A total of around 92% of correct predictions were obtained through an independent validation set (table S1).

Table S1. Confusion matrix for the best PLS-DA model based on the whole MIR spectra pre-processed with SNV followed by SG (15,2,2), using 3 LV and considering only the independent testing data set. The values are expressed in percentage.

	Predicted classes	
Real classes	GL25	Control
GL25	41.7%	8.3%

	(5/6)	(1/6)
Control	0%<br>(0/6)	50.0%<br>(6/6)

	Predicted classes	
Real classes	GL25	Control
GL25	5/6	1/6
Control	0/6	6/6

Besides the percentage of correct predictions, confusion matrices provide insight into which classes were best predicted and which were misclassified. The respective confusion matrix shows that control was accurately predicted, with 100% of correct predictions. Regarding GL25, 83.3% of the samples were correctly predicted, while 16.7% were misclassified as belonging to control. These results reinforce the abovementioned findings from spectral analysis and PCA, which indicated differences between these two classes.

The sensitivity and specificity values are shown in table1.

Table S2. Sensitivity and specificity values calculated according to equations 1 and 2 for the best PLS-DA model.

**Classes**	**Sensitivity**	**Specificity**
**GL25**	0.833	1
**Control**	1	0.833

GL 25 presents sensitivity and specificity values of 0.833 and 1, respectively, while control presents sensitivity and specificity values of 1 and 0.833, respectively. These values demonstrate the accuracy of the PLS-DA model.

Overall, the PLS-DA results revealed that the MIR spectra of embryonic chicken femur can be used to discriminate between these two classes with very good accuracy.

The wavenumbers that showed the highest importance for the developed model can be seen through the analysis of the regression coefficient vectors Fig. S3.

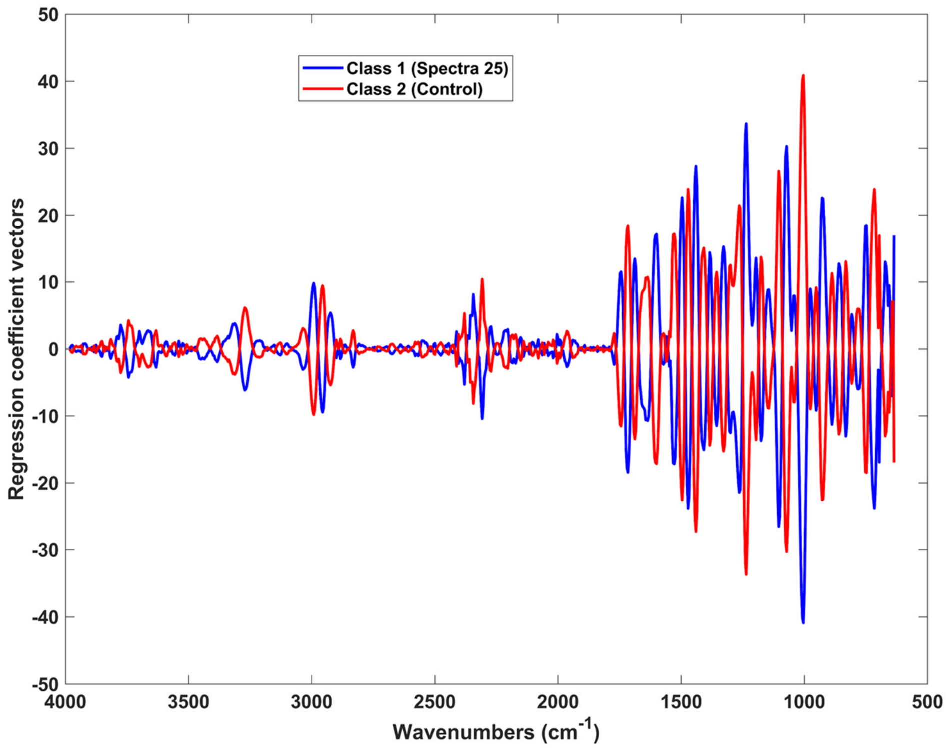

Fig. S3. Regression coefficient vectors of the best PLS-DA model obtained from MIR spectra when pre-processed with Savitzky-Golay filter (15 points filter width, 2nd polynomial order and second derivative) followed by mean-centering.

The most important wavenumbers for all the classes were located between 1,700 and 1,000 cm^{-1}. This is in agreement with another interesting study, that stated that the spectral interval that allowed the classification of five different types of collagen using MIR spectra was within 1,700 and 1,005 cm^{-1} [j]. Theoretically, the biggest differences between MIR spectra of GL25 and control samples were expected due to the glycation of proteins and collagen cross-linking . The glycation of proteins is expected to be seen more strongly around 1,200 to 1,000 cm^{-1} as referred by Khajehpour and co-authors [35]. In fact, through the analysis of the regression coefficient vectors this was not clear as all the regions within 1,700 and 1,000 cm^{-1} showed an important contribution to the developed PLS-DA model. However, in figure, which depicted the average raw spectra of both classes, it is visible that GL25 spectra showed a more prominent band around these wavenumbers. This reinforces the idea that this class shows a higher glycosylation than the control as expected. Regarding collagen cross-linking, some articles referred that the wavenumbers around 1,660 and 1,690 cm^{-1} may reflect structural cross-linking changes [62,63]. Therefore, these findings support why the most important wavenumbers for the PLS-DA model were located within 1,700 and 1,000 cm^{-1}.
